# Predicting fear of COVID-19 based on depression, anxiety, and stress in administrative employees by emphasizing the role of ethnicity

**DOI:** 10.1186/s12879-025-11485-2

**Published:** 2025-09-19

**Authors:** Mohammad Taghi Badeleh Shamushaki, Elaheh Sadeghi, Javad Rahmani

**Affiliations:** 1 Golestan University of Medical Sciences, Gorgan, Iran; 2 Golestan University of Medical Sciences, Gorgan, Iran; 3 Department of Psychology, Islamic Azad University, Go.C, Gorgan, Iran

**Keywords:** Fear of COVID-19, Stress, Anxiety, Depression, Ethnicity

## Abstract

**‌Background:**

The COVID-19 pandemic has generated profound global mental health challenges, yet the interplay between depression, anxiety, stress, ethnicity, and fear of COVID-19 remains underexplored in occupational contexts. This study examines the prediction of COVID-19 fear through depression, anxiety, and stress, with particular attention to ethnic differences among Iranian administrative employees.

**Methods:**

A cross-sectional analysis was conducted with 524 participants from three ethnic groups (Fars, Turkmen, Mazandarani) recruited through convenience sampling from administrative offices in Gorgan, Iran. Standardized instruments (DASS-21, Fear of COVID-19 Scale) assessed psychological variables. Hierarchical regression and moderation analyses were performed using SPSS 22, with multicollinearity diagnostics (VIFs < 2.55) and reliability metrics (α = 0.843–0.917).

**Results:**

Anxiety (β = 0.324, *p* < 0.001) and stress (β = 0.182, *p* = 0.004) significantly predicted COVID-19 fear explained 19.9% of the variance. Depression showed no significant predictive role despite a moderate bivariate correlation (*r* = 0.315). Crucially, no ethnic differences emerged in fear levels (*p* = 0.314), nor did ethnicity moderate psychological predictors (ΔR² = 0.001, *p* = 0.547). Women reported significantly higher fear than men (*p* < 0.05).

**Conclusion:**

While anxiety and stress are critical predictors of fear of COVID-19, the absence of ethnic disparities suggests that occupational homogeneity may attenuate ethnocultural differences in mental health outcomes during health crises. Workplace interventions targeting anxiety and stress reduction are strongly recommended, particularly for female employees.

## Background

The COVID-19 pandemic has precipitated unprecedented disruptions to global societal functioning, extending beyond physical health threats to inflict profound psychological consequences. By February 2021, the World Health Organization (WHO) documented over 113 million confirmed infections and 2.5 million fatalities worldwide [1–3]. Critically, the pandemic’s impact transcends morbidity and mortality metrics, manifesting in well-documented escalations of stress, anxiety, depression, sleep disturbances, and occupational burnout [4–7].

This crisis presents a dual dynamic: while COVID-19 undermines mental health, psychological resilience conversely shapes pandemic responses. Notably, pandemic-related uncertainty can inflict greater societal damage than the virus through maladaptive panic [5]. Meta-analytic Evidence confirms that fear of COVID-19 exhibits moderate associations with depression (20 studies) and strong linkages to anxiety (24 studies) [7], influencing critical domains including health decision-making, coping strategies, and lifestyle behaviors.

Fear of disease transmission constitutes a cardinal psychological response to pandemics, serving evolutionarily adaptive functions when proportionate to threat [8]. Historically observed during Ebola and HIV/AIDS outbreaks, fear mobilizes protective behaviors during health crises [8]. However, when exceeding rational thresholds, it precipitates: Heightened anxiety and social withdrawal, Functional impairment in daily activities, Exacerbation of pre-existing psychiatric conditions, and Maladaptive coping (e.g., substance abuse, obsessive rituals) [5, 8–10].

Studies demonstrate bidirectional relationships between COVID-19 fear and mental health comorbidities. While anxiety amplifies fear-driven avoidance behaviors [11], appropriately modulated fear can promote preventive health actions [8, 11]. Conversely, chronic stress compounded by isolation fuels pathological mental states including PTSD, suicidal ideation, and affective dysregulation [5, 12].

Fear determinants are multifactorial, emerging from: (1) Health apprehensions (personal or familial vulnerability). (2) Socioeconomic stressors (employment precarity, financial instability) [10, 13, 14]. (3) Structural inequities shaping differential exposure and coping resources [15].

Demographic moderators further diversify fear manifestations; Women report elevated fear despite lower mortality, suggesting gendered vulnerability patterns [16, 17]. Age: Younger cohorts exhibit heightened fear relative to resilient elders [10, 18]. Ethnicity; Marginalized groups experience amplified fear due to healthcare barriers and economic disadvantage [19–21].

These disparities persist despite declining mortality, with racial minorities enduring compounded stressors from intersecting discrimination and resource limitations [16, 20, 21]. Crucially, the behavioral effects of fear, such as adherence to health guidelines, are influenced by cultural backgrounds and past experiences with healthcare [22, 23].

Golestan Province (Iran) presents elevated baseline mortality (7.02/1,000) with gendered differentials (males: 7.12; females: 6.10/1,000), reflecting occupational exposures and pre-existing cardiopulmonary disease burdens that potentiate COVID-19 severity [24]. In this area, the ethnic diversity of Gorgan—especially the coexistence of Fars and Turkmen—offers a natural setting to investigate how cultural frameworks, health beliefs, and socioeconomic status influence psychological responses.

This study therefore investigates anxiety, stress, and depression as predictors of COVID-19 fear among Iranian administrative employees, with particular attention to ethnocultural moderators that may elucidate differential vulnerability patterns during public health crises.

## Methods

### Participants and procedures

This cross-sectional study was conducted with the participation of 524 employees aged 20 to 60 from government offices in Gorgan, Iran. Participants belonging to three ethnic groups — Fars, Turkmen, and Mazandarani — were recruited through the convenience sampling method. We used Morgan’s table to determine the sample size. Sample size determination followed the Krejcie & Morgan’s (1970) formula for finite populations: *n* = (χ² N P (1-P)/(d² (N-1) + χ² P (1-P). Accounting for 20% attrition risk (common in pandemic field studies), the target sample was 440. Our final sample (*N* = 524) exceeded this threshold, providing adequate power for subgroup analyses (Fars: *n* = 327; Turkmen: *n* = 149; Mazandarani: *n* = 48).

The inclusion criteria consisted of being employed in the selected government offices and willingness to participate in the study by providing informed consent. The criteria for exclusion involved incomplete questionnaires and withdrawal from the study at any point, along with the removal of individuals with psychiatric conditions such as major depressive disorder, personality disorders, obsessive-compulsive disorder, and substance use disorder from this research. These criteria were applied to ensure data integrity and the effective participation of the participants. All ethical principles, including confidentiality, professional ethics, and voluntary participation, were strictly adhered to in this study.

### Measures

Demographic Checklist: This checklist included demographic variables such as gender, age, education level, marital status, history of specific illnesses, native language, and the duration of the study related to COVID-19 awareness.

DASS-21: Developed by Lovibond and Lovibond [25], this questionnaire measures three psychological constructs—depression, anxiety, and stress—using 21 items (7 items for each construct). Scoring is based on a 4-point Likert scale (from 0 to 3). Previous studies reported the reliability of this questionnaire with a Cronbach’s alpha coefficient of 0.81. Also, in this study, reliability analysis shows DAS-21 (Depression/Anxiety/Stress): α = 0.917 (superb).

Fear of COVID-19 Scale: This 7-item scale, developed by Ahorsu et al. [26], measures fear of COVID-19 using a 5-point Likert scale (1 = strongly disagree to 5 = strongly agree). The score range for this scale is between 7 and 35, with higher scores indicating higher levels of fear. The reliability of the original version was with a Cronbach’s alpha coefficient of 0.82 and a test-retest reliability of 0.88. Additionally, the reliability analysis conducted in this study reveals that the Fear of COVID-19 Scale has an α = 0.843 (indicating excellent reliability).

### Data analysis

Using SPSS software (version 27), descriptive statistics, including the mean and standard deviation, were computed for all variables. Then, Pearson’s correlation coefficient was used to examine the relationships between the psychological variables (stress, anxiety, and depression) and fear of COVID-19. Analysis of variance (ANOVA) was used to analyze differences among ethnic groups and demographic subgroups. Ultimately, multiple regression analysis was employed to assess how psychological factors predict fear of COVID-19 and to explore the moderating influence of ethnicity.

## Results

The study included 524 Iranian adults (71.0% male; 29.0% female). Key demographic findings: Age: M = 40.21 (SD = 7.72), range = 21–63, Marital Status: 88.1% married, Education: 87.9% Bachelor’s degree or higher, Ethnicity: Fars (62.4%), Turkman (28.4%), Mazandarani (9.2%).

Demographic and psychological characteristics stratified by ethnicity are presented in Table 1. All three ethnic groups (Fars, Turkmen, Mazandarani) showed comparable levels of depression (M ≈ 4.48–4.63), anxiety (M ≈ 3.77–3.85), and stress (M ≈ 6.15–6.67). Fear of COVID-19 scores were similarly distributed across groups (Fars: M = 16.86; Turkmen: M = 16.66; Mazandarani: M = 15.35), with no clinically significant variations. Standard deviations indicate moderate within-group variability, particularly for depression (SD ≈ 4.00) and fear (SD ≈ 5.50).

**Table 1:** Participant characteristics in ethnicity

Ethnicity		Depression	Anxiety	Stress	Fear of COVID-19
Fars	*N*	327	327	327	327
	Mean	4.4771	3.8502	6.6667	16.8593
	Std. Deviation	3.99830	3.79524	4.45587	5.50057
Turkman	*N*	148	149	149	149
	Mean	4.6284	3.7919	6.1544	16.6644
	Std. Deviation	4.00218	3.89087	4.41546	5.40347
Mazandarani	*N*	48	48	48	48
	Mean	4.5625	3.7708	6.4583	15.3542
	Std. Deviation	4.27216	4.30852	4.62168	5.67372
Total	*N*	523	524	524	524
	Mean	4.5277	3.8263	6.5019	16.6660
	Std. Deviation	4.01776	3.86414	4.45693	5.49510

Every measure showed a departure from normality (K-S *p* < 0.001), justifying the application of nonparametric tests when appropriate; however, the substantial sample size supports the validity of parametric tests. Intercorrelations among key variables are detailed in Table 2. Anxiety and stress showed the strongest association (*r* = 0.707, *p* < 0.001). Fear of COVID-19 correlated moderately with both anxiety (*r* = 0.430) and stress (*r* = 0.387).

**Table 2:** Correlation matrix

Variable	1	2	3	4
Fear of COVID-19	**-**			
Anxiety	0.430	**-**		
Depression	0.315	0.672	**-**	
Stress	0.387	0.707	0.718	**-**

Hierarchical regression coefficients predicting COVID-19 fear appear in Table 3. Anxiety emerged as the strongest predictor (β = 0.324, *p* < 0.001). Stress contributed significantly but less robustly (β = 0.182, *p* = 0.004). Depression showed no predictive value (β = −0.033, *p* = 0.580) despite bivariate correlations. Multicollinearity was negligible (all VIFs < 2.55). Primary Regression: Predicting Fear of COVID-19, Model Summary: *R²* = 0.200, F (3,519) = 43.14, *p* < 0.001, 19.5% variance explained. Anxiety contributed 78% more to Fear of COVID-19 than stress (β ratio: 0.324/0.182). Depression was non-significant despite bivariate correlation (*r* = 0.315), suggesting suppression effects.

**Table 3:** Regression analysis for predicting fear of COVID-19

Variable	β	95% CI	*p*-value	VIF
Anxiety	0.324	[0.30, 0.63]	< 0.001	2.25
Stress	0.182	[0.07, 0.38]	0.004	2.54
Depression	− 0.033	[−0.21, 0.12]	0.580	2.32

The results of the moderation analysis comparing different models are presented in Table 4.

**Table 4:** Moderation model comparison

Model	Predictors	*R*²	ΔR²	*p*-value
1	Anxiety, Stress, Depression	0.200		< 0.001
2	+ Ethnicity Interaction	0.200	0.001	0.547

Adding ethnicity interactions explained negligible incremental variance (Δ*R²* = 0.001). The non-significant interaction (*p* = 0.547) confirms ethnicity did not moderate psychological predictors. Step 1 (Main Effects): *R²* = 0.200. Step 2 (+ Ethnicity Moderation): Δ*R²* = 0.001, F (1,518) = 0.36, *p* = 0.547. Interaction Effect: β = 0.034, *p* = 0.547. Ethnicity did not moderate relationships between Anxiety, Stress, Depression, and Fear of COVID-19.

Multicollinearity: All VIFs < 2.55 (tolerance > 0.39), Eigenvalues > 0.09 (condition indices < 6.54), Outliers: 7 cases identified | Std. Residuals > |2.5|, Cook’s D < 0.02 (no influential cases), Residuals: Durbin-Watson = 2.11 (no autocorrelation). Furthermore, analyses were conducted to examine the effects of gender, age, education level, and marital status, with the following results: Gender: Women reported higher levels of fear of COVID-19 than men (*p* < 0.05). Age: No significant differences in fear of COVID-19 were observed among age groups (*p* > 0.05). Education: The level of education did not significantly influence fear of COVID-19 (*p* > 0.05). Marital Status: No significant differences in fear of COVID-19 were found between single and married individuals (*p* > 0.05).

## Discussion

This research focuses on the impact of ethnicity and explores the factors influencing COVID-19-related fear among administrative workers in Iran. Key findings confirm anxiety (β = 0.324, *p* < 0.001) and stress (β = 0.182, *p* = 0.004) are significant predictors of fear, consistent with global literature linking pandemic-related distress to fear responses [3, 27, 28]. Notably, depression showed no predictive value despite a moderate bivariate correlation (*r* = 0.315), suggesting its relationship with fear may be mediated by anxiety/stress or suppressed in multivariate models—a finding warranting further investigation.

The regression model explained 19.9% of the variance in fear, underscoring the contribution of anxiety/stress while highlighting unmeasured factors (e.g., media exposure, socioeconomic status) as critical gaps. This finding aligns with pandemic literature where contextual variables (e.g., local outbreak severity and policy changes) significantly modulate fear [7, 10]. To address this, we contextualized our data collection period (May–July 2020) within Golestan Province’s Delta-variant surge [24], though future studies should directly integrate such variables.

This finding is in agreement with previous literature [3, 6, 29, 30], which indicates that fear of COVID-19 is more strongly associated with anxiety and stress and less so with depression [27]. Therefore, a comprehensive understanding of the prevalence of mental health issues during the COVID-19 pandemic across different populations is needed so that policymakers can implement effective countermeasures [3, 31]. Fear of contracting or transmitting COVID-19, known as fear of the virus itself or its spread through close contact, is a key factor that can lead to increased levels of stress and anxiety during the pandemic [32]. It has been proven that fear of infection may exacerbate pre-existing mental health disorders or trigger severe anxiety responses [31].

Contrary to studies reporting ethnic disparities in COVID-19 fear [18–20, 33], no significant differences emerged among Fars, Turkmen, or Mazandarani groups (*p* = 0.314), nor did ethnicity moderate psychological predictors. The results may reflect our sample’s unique characteristics: (1) Occupational homogeneity: Administrative employees likely share comparable job security, healthcare access, and COVID-19 information channels, potentially attenuating ethnic disparities. (2) Regional equity: Gorgan’s integrated healthcare infrastructure may reduce ethnic gaps in pandemic-related stressors [24]. (3) Measurement limitations: Treating ethnicity as a categorical variable without assessing sociocultural mediators (e.g., discrimination, health beliefs) risks oversimplification.

Some studies have shown that specific ethnic groups, particularly racial and economic minorities in Western countries, experience a higher level of fear and stress due to limited access to healthcare and experiences of discrimination [27]. The findings suggest that other variables, such as discrimination, social pressures, and access to supportive resources, may influence the experience of fear of COVID-19 [34]. Similar patterns of discrimination have been observed in the experiences of Asian-Americans, and associating the origin of the virus with these groups has led to increased stigma and fear [35]. It’s crucial to recognize that existing health issues are negatively correlated with fear, highlighting the necessity for a precise understanding of these elements [36].

Understanding how ethnic factors relate to fear of COVID-19 can improve public health strategies and culturally sensitive interventions. The effects of fear are not only associated with immediate psychological outcomes but may also have long-term consequences on community health and resilience to future health crises [37]. However, this study observed that administrative employees in Gorgan, regardless of ethnicity, reported similar levels of fear and anxiety. This finding may be attributed to the relatively similar socio-economic conditions of these groups and more equal access to information and healthcare services in administrative environments.

We also found that women reported higher fear, consistent with global trends of heightened health anxiety among females [16, 37]. However, age, education, and marital status showed no significant effects—contrasting with studies linking older age or lower SES to greater fear [10, 17]. This discrepancy might be due to the limited variety of occupations in our sample. This finding is in line with the studies indicating that women, due to greater sensitivity to environmental stressors and health-related concerns, experience a higher level of anxiety and fear in the face of health crises [16, 17, 38].

In contrast, age, education level, and marital status did not have a significant effect on fear of COVID-19. The persistent impact of COVID-19 on mental health underscores its critical role in combating the pandemic. These results highlight the importance of psychological interventions to reduce stress and anxiety during health crises and suggest that health policymakers should develop programs to support the mental health of employees, particularly at-risk groups such as women.

## Conclusion

This study investigated the psychological predictors of fear of COVID-19 among administrative employees, with particular attention to the potential role of ethnicity. The results revealed that higher levels of anxiety and stress significantly predicted greater fear of COVID-19, whereas depression and ethnicity were not significant predictors. These findings indicate that anxiety and stress are more salient than depressive symptoms in shaping individuals’ fear responses during a pandemic context.

Contrary to expectations, ethnicity did not significantly influence fear levels, suggesting that within this occupational and cultural sample, fear of COVID-19 may be shaped more by other factors than by ethnocultural background. This finding highlights the importance of psychological screening and tailored interventions targeting anxiety and stress management in workplace mental health programs, especially in the face of ongoing or future public health emergencies.

However, it is essential to recognize various limitations. The cross-sectional design precludes any causal inference and limits understanding of temporal relationships between psychological distress and fear. The use of self-report instruments raises concerns about potential bias due to social desirability or inaccurate self-assessment. Moreover, the study sample was restricted to administrative employees, which may limit the generalizability of the findings to broader or more diverse populations. Additionally, ethnicity was treated as a categorical variable without accounting for nuanced sociocultural or identity-related dimensions that could interact with fear responses to health threats.

Future research should consider longitudinal methodologies to explore causal pathways, include more heterogeneous and representative samples, and integrate qualitative or mixed-methods approaches to better capture the complex interplay between cultural identity, psychological distress, and pandemic-related fear.

## Data Availability

The datasets generated and analyzed during this study are not publicly available due to confidentiality agreements and ethical restrictions protecting participant privacy. However, the data may be made available upon reasonable request, subject to institutional approval. Interested researchers should direct inquiries to the corresponding author at [badeleh@gmail.com] for access.

## Abbreviations

COVID-19: Coronavirus disease 2019
WHO: World Health Organization
HIV/AIDS: Human immunodeficiency virus /acquired immunodeficiency syndrome
DASS-21: Depression, Anxiety, Stress scales- 21
